# A Comparative Numerical Study on the Performances and Vortical Patterns of Two Bioinspired Oscillatory Mechanisms: Undulating and Pure Heaving

**DOI:** 10.1155/2015/325934

**Published:** 2015-05-11

**Authors:** Mohsen Ebrahimi, Madjid Abbaspour

**Affiliations:** Department of Mechanical Engineering, Sharif University of Technology, Tehran 9567-11155, Iran

## Abstract

The hydrodynamics and energetics of bioinspired oscillating mechanisms have received significant attentions by engineers and biologists to develop the underwater and air vehicles. Undulating and pure heaving (or plunging) motions are two significant mechanisms which are utilized in nature to provide propulsive, maneuvering, and stabilization forces. This study aims to elucidate and compare the propulsive vortical signature and performance of these two important natural mechanisms through a systematic numerical study. Navier-Stokes equations are solved, by a pressure-based finite volume method solver, in an arbitrary Lagrangian-Eulerian (ALE) framework domain containing a *2D NACA0012* foil moving with prescribed kinematics. Some of the important findings are (1) the thrust production of the heaving foil begins at lower St and has a greater growing slope with respect to the St; (2) the undulating mechanism has some limitations to produce high thrust forces; (3) the undulating foil shows a lower power consumption and higher efficiency; (4) changing the Reynolds number (Re) in a constant St affects the performance of the oscillations; and (5) there is a distinguishable appearance of leading edge vortices in the wake of the heaving foil without observable ones in the wake of the undulating foil, especially at higher St.

## 1. Introduction

The hydrodynamics and energetics of bioinspired oscillating mechanisms have received significant attentions by engineers and biologists over the past decades. Regarding the development of biomimetic robots, autonomous underwater vehicles (AUVs), and Micro Air Vehicles (MAVs), efficient control surfaces and propulsion mechanisms are required to produce propulsive forces and/or moments [1]. The oscillating mechanisms which are used by animals in nature can provide forces and moments efficiently [2–4]. This is due to the evolution process of these animals and their adaptation with operating medium [5]. Heaving (or plunging) and undulation are two significant mechanisms which are commonly used by body and/or fins of aquatic animals and wings of aerobic animals for propulsion, maneuvering, and stabilization purposes [2]. For example, in some fish species (some kinds of carangiform and Thunniform swimmers, e.g., some of the dolphins, tunas, or sharks), the caudal fin has pure heaving motions, while in some other species (anguilliform and subcarangiform swimmers like spinny dogfish shark) it has undulating motions [5, 6].

In propulsion, flying animals generally use oscillatory mechanisms to generate both thrust and lift, while many swimming species use oscillatory mechanisms primarily to generate thrust [7]. Animals moving in water have usually either longitudinally slender (thin) or vertically (rarely laterally) flat body and caudal fins, which gives them a higher flexibility for propulsion, a lower drag to move against, and, hence, less need for exertion [2]. In many species, caudal fin provides whole or a bulk of the propulsive, maneuvering, and stabilizing forces. The caudal fin of these animals has a foil-like geometry in 2D cross sections. An oscillating (rigid heaving or flexible undulating) caudal fin produces thrust under certain conditions [8]. The thrust is produced through the formation of an average velocity profile behind the oscillating fin which is in the form of a jet [9]. Because of the influence of the periodic excitation from the fin, a downstream moving staggered array of vortices, closely resembling a Karman vortex street but with reverse rotational direction, is formed in the wake [9]. The physics of thrust generation with oscillating motions was first presented by Durand [10] and after that observed in a number of experimental studies, for example, [11–13]. Rosen [14], Videler et al. [15], Lauder [5, 16], Drucker and Lauder [17], and Tytell and Lauder [18] are also some of the researchers who observed experimentally similar patterns in the wake of different fish species. However, numerical studies have provided more details on wake structures and effective parameters of undulatory motion of flexible body or fins [19–22] as well as vortical pattern in oscillating heaving foils [7, 9, 23–25].

Freymuth [11], Koochesfahani [12], Jones et al. [25], and Zhang et al. [26] showed through experiments and simulations that the wakes of oscillating airfoils can be characterized as drag-producing, neutral, or thrust producing mechanisms depending on the heaving/undulating frequency and amplitude. The general conclusion from the existing investigations is that the thrust force and propulsive efficiency strongly depend on the kinematic parameters of oscillations. Furthermore, the wake structure of the oscillatory mechanisms is closely related to the nondimensional parameter Strouhal number (St = 2*fA*/*U*), where *U* is the free-stream velocity, *A* is the amplitude of oscillations, and *f* is the oscillating frequency.

In this paper a* NACA0012* foil, which oscillates with heaving and undulating mechanisms, is studied in three low, medium, and high Reynolds numbers, respectively, *Re* = 4000, *Re* = 40000, and *Re* = 400000 (*Re* = *UL*/*ν*, where *L* is the characteristic length equal to the chord length of the foil and *ν* is the kinematic viscosity of the fluid). The oscillating frequency is varied from 0.001 to 0.05 for *Re* = 4000, from 0.01 to 0.5 for *Re* = 40000, and from 0.1 to 5 for *Re* = 400000, which are equivalent to the St in the range of [0.05–2.5] with maximum amplitude excursion of 0.1*L*. In present study, simulations are carried out for a typical foil geometry (*NACA0012*) which is commonly used in many applications as control surfaces or propulsors. In addition, a number of tests are accomplished to validate and evaluate the numerical solver in comparison with experimental data.

Several experimental and numerical works have separately studied the wake structure, energetics, and flow characteristics of undulating and heaving foils including the lift, drag, and propulsion efficiency [12, 13, 26–35]. However, this study attempts to present a comparison for the energetics, performance, and wake structures of the heaving and undulating mechanisms in an extended range of Strouhal numbers. Such comparisons could provide a wealth of data and consequences about the advantages and disadvantages of these mechanisms and also are helpful to select and design the appropriate mechanism for the specific applications. Furthermore, remarkable findings are brought out from this comparison which are presenting in Section 4. The range of Strouhal numbers reported here, that is, 0.05 < St < 2.5, for the undulating motion is wider than the well-known range in literature, that is, 0.2 < St < 0.4 [36]. This is in order for more observations and answering the following questions: why do the animals use the undulatory propulsion mechanisms in the reported range? And why do not they use lower or higher ranges of Strouhal numbers? In addition, the presented qualitative and quantitative data are useful in the designing and developing of bioinspired oscillatory mechanisms. Current research also brings out a comparison of the especial deformability of the undulating fins over the rigidity of the heaving fins which mostly exist in aquatic animals. Owing to the difficulty in testing the swimming parameters of live fish, experimental studies on the oscillating caudal fins are rare and hence such systematic numerical studies which can simulate the real conditions are essential.

The rest of this paper is organized as follows. The governing equations as well as methodology and implementation of numerical approach, including the enforcement of boundary conditions and the mesh motion and correction procedure, are described in the next section. In addition, the validation tests and calculation of parameters are presented. Then, the results including thrust, consumed power, efficiency, and wake structure of the heaving and undulating mechanisms are presented and discussed in detail. Finally, the major conclusions are presented.

## 2. Materials and Methods

### 2.1. Fluid-Solid Interaction Problem

The oscillations of bodies such as heaving and undulation of foils involve moving and deforming boundaries, respectively. The arbitrary Lagrangian-Eulerian (ALE) approach [37] is employed to encounter the fluid and moving body interactions. From the ALE viewpoint, the nodes of the computational domain mesh may be moved with the continuum, same as normal Lagrangian manner, be held fixed in Eulerian fashion, or be moved in some arbitrarily specified way to give a continuous rezoning capability [38]. In addition, the simulation of conservation equations on the moving meshes requires the geometric conservation law (GCL) to be satisfied [39].

### 2.2. Governing Equations of Fluid

The ALE formulations of the unsteady incompressible viscous flow, governed by the integral forms of Navier–Stokes equations, are as follows.

Conservation of mass:

(1) $$\begin{array}{c} \frac{\partial }{\partial t}\overset{}{\underset{V\left(t\right)}{\int }}\rho V\left(\;t\;\right)+\overset{}{\underset{S\left(t\right)}{\int }}\rho \left(\;\vec{v}-{\vec{v}}_{g}\;\right)\cdot \hat{n}\;dS=0. \end{array}$$

Conservation of momentum:

(2) ∂∂t∫Vtρv→ dV+∫Stρv→v→−v→g·n^ dS=∫Vt∇·σ+ρf→bdV,

where, in the above equations, *ρ* is the fluid density, $\vec{v}$ is the Cartesian velocity vector of the fluid, *V*(*t*) is an arbitrary volume whose boundary *S*(*t*) = ∂*V*(*t*) moves with the mesh velocity ${\vec{v}}_{g}$, ${\vec{f}}_{b}$ is the specific body force vector, and *σ* denotes the Cauchy stress tensor which can be calculated from

(3) $$\begin{array}{c} \sigma_{ij}=-p\delta_{ij}+\tau_{ij}, \end{array}$$

where *p* = (*τ*_11_ + *τ*_22_ + *τ*_33_)/3 is the thermodynamic pressure, *δ*_*ij*_ is the Kroenecker delta function, and *τ*_*ij*_ are dynamic stresses (shear and normal stresses) which for an incompressible, isotropic, Newtonian fluid are expressed as

(4) $$\begin{array}{c} \tau_{ij}=\mu \left(\;\frac{\partial v_{i}}{\partial x_{j}}+\frac{\partial v_{j}}{\partial x_{i}}\;\right), \end{array}$$

where *μ* is the dynamic viscosity of the fluid.

The two important nondimensional numbers in this problem are Reynolds number (Re), which is the ratio of inertial over viscous forces and characterizes steady inline motion of heaving and undulating foils, and Strouhal (St) number which is the ratio of unsteady to inertial forces and describes the structure of wakes (or vortical patterns) behind the heaving and undulating foils which oscillate with frequency *f* (or period *T*) [40, 41].

### 2.3. Simulation Algorithm

**Figure 1:**
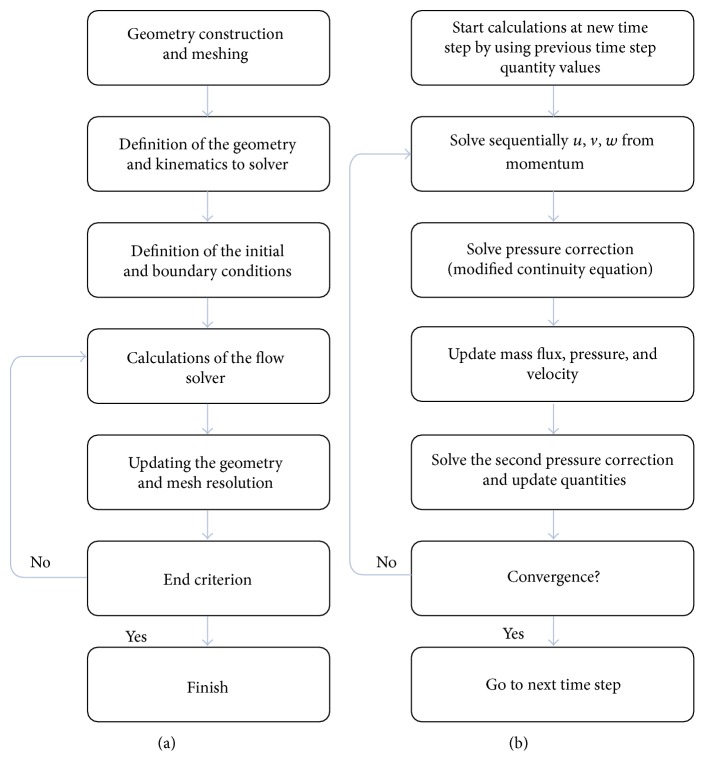
(a) The fluid-solid interaction problem algorithm. (b) Flowchart of fluid solver.

The overall procedure for carrying out the computational simulations of the fluid-solid interaction problem of oscillating foil can be divided into the following major steps (Figure 1(a)):

- Construction of the geometry and generating the mesh for foil and computational domain.
- Defining the geometry and kinematics into the solver.
- Defining the appropriate initial and boundary conditions.
- Performing the unsteady CFD computations and obtaining required hydrodynamic forces.
- Updating the motion or deformation of the foil's geometry and moving or modifying the mesh with dynamic mesh resolution techniques.
- Continuing the unsteady solution until steady periodical drag, lift, and moment values are observed or more periods of oscillations.

### 2.4. Solver

The implicit method is conducted for the temporal discretization of the equations. As well, the second-order upwind scheme is utilized for the convective fluxes which require the determination of the gradient of scalar properties [42]. Also, the least squares scheme is used in the solver for the gradient evaluation associated with the Total Variation Diminishing (TVD) limiter [43, 44] to prevent numerical oscillations. In addition, a second-order accurate, central-difference approximation is applied to the diffusion terms (dynamic stresses) in momentum equations. The collocated grid approach is employed in which all of the flow variables are stored at the same locations and thus, the number of coefficients that must be computed and stored is minimized. The pressure-velocity coupling is achieved by utilizing the Pressure-Implicit with Splitting of Operators (PISO) scheme [45] which, by using a relationship between velocity and pressure corrections, enforces the mass conservation and obtains the pressure field. Furthermore, a segregated pressure-based solver is employed to sequentially solve the governing equations.  Figure 1(b) illustrates the flowchart of the fluid solver which is outlined as follows:

- Starting the calculations at the new time step using the latest solution of velocity components and pressure field as starting estimates for current time.
- Sequentially solving the momentum equations by utilizing the values of the pressure and face mass fluxes from previous time step.
- Solving the pressure correction equation using the recently obtained velocity field and the mass flux.
- Correction of face mass fluxes, pressure, and the velocity field using the pressure correction obtained from previous step.
- Solving the second pressure correction equation and correcting both velocities and pressure again.
- Checking for the convergence of the equations; if the convergence was not reached returning to step 2 and repeating; otherwise, proceeding to next time step.

The convergence criterion is conducted so that the Root Mean Squares (RMS) of the pressure and the velocities are less than 10^−5^ in the computational domain nodes.

### 2.5. Mesh Construction

**Figure 2:**
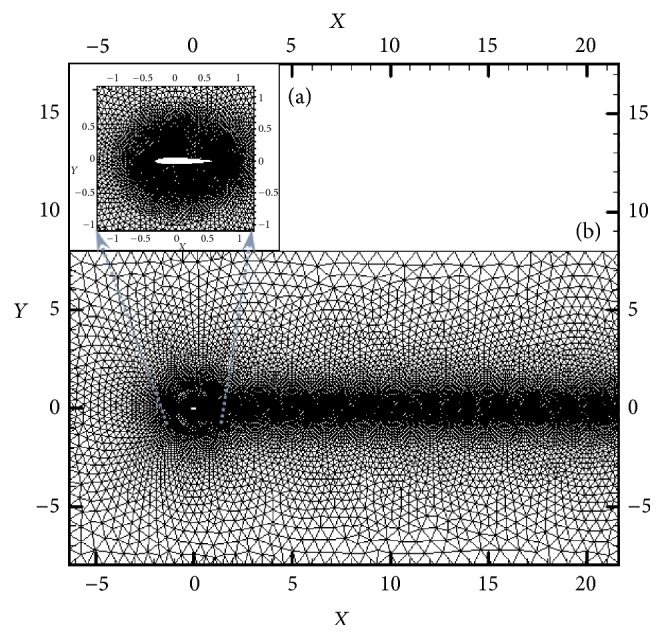
*NACA0012* foil and (a) the refining region of circle shape; (b) the computational domain.

Combined structured and unstructured meshes of 20436 nodes and 30535 elements are constructed around the* NACA0012* foil (Figure 2) and also for the validation purposes, two other meshes with the same geometrical constructions are constructed by approximately half and twice the number of grid nodes of the mentioned mesh. The physical phenomena of fluid mechanics such as boundary layer and velocity and pressure gradients are essential factors to the construction of the mesh. The laminar boundary layer thickness is estimated with Blasius equation [46]. In order to decrease the computational costs of dynamic mesh refinements (as are described in coming sections), the solution domain is composed of two parts: a nondeforming region which remains unchanged during the deformations (Figure 2(b)) and a deforming region (circle) which is altered by the flexible motions of the foil (Figure 2(a)).

### 2.6. Boundary Conditions

A limited solution domain is created around the body, whose boundaries are far enough to not affect the results at far-field boundaries, particularly the velocity field and the wake structure. The foil is located at 6*L* from the inlet in the axial direction (*L* is the chord length equal to 1 m), 8*L* from each transverse far-field, and 20*L* from the outlet. Simulations are carried out in *Re* = 4000, 40000, and 400000 for St in the range of [0.05, 2.5] to study the variations of instantaneous and time averaged forces, consumed power, efficiency, and flow patterns versus St. Inlet velocity, outlet pressure, and far-field free-stream velocity are prescribed. The inner oscillating zone and the outer steady zone in the domain mesh are created with a common overlapping interface describing an interior zone.

### 2.7. Mesh Motion and Correction Techniques

The foil deforms flexibly by undulation motions and makes the surrounding flow strongly unsteady. In addition, the computational grids are rigidly moved with the heaving motions. To simulate this unsteady flow, dynamic grid techniques should be employed along with the corresponding unsteady flow solver. The boundary-conforming methods (dynamic grid methods) are still more popular in CFD community including the simulations of the external biofluid dynamics [26]. The mostly used approach in this category is “spring-analogy-method” (SAM) [47], which is an algorithm to move the grid nodes surrounding the deforming solid walls supposing the cell edges as springs. For large deformations, other mesh refinement methods such as adaptive local refinement [48, 49] should be used accompanied with SAM to resolve the mesh distortion problems. On the other hand, rigid-body motions use the body fitted mesh approach [50] to impose the translational motions of heaving in such a way that whole domain translates with the translations of the rigid boundary.

### 2.8. Kinematics

**Figure 3:**
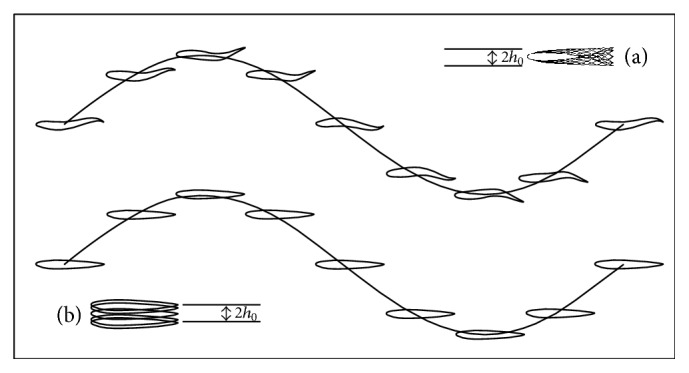
The kinematics of (a) the undulatory motion; (b) the heaving motion.

The kinematic formulations of heaving motions are conducted as (5) (Figure 3(a)). Consider

(5) $$\begin{array}{c} y_{pl}\left(\;x,t\;\right)=y+A_{pl}\sin\left(\;2\pi ft\;\right), \end{array}$$

where *y*_*pl*_ is the moved *y*-coordinate of the point (*x*, *y*) because of heaving with amplitude *A*_*pl*_. In all of the simulations, the normalized maximum heaving amplitude *A*_*pl*_ is 0.1, in accordance with the maximum excursion of the undulating motions.

The formulations of the undulatory motions for the chord line or the backbone of the virtual fish are as (6) (Figure 3(b)). Consider

(6) yx,tAxsin⁡2πλx−2πft,Ax=a0+a1x+a2x2

with the coefficients *a*_0_ = 0.02, *a*_1_ = −0.08, and *a*_2_ = 0.16 to match the experimental curve of a typical carangiform swimmer [21, 51]. In all of the simulations, the normalized wavelength *λ*/*L* is 1.

### 2.9. Wake Visualization

The most important characteristics of the flow are location of the vortex cores and distribution and breakdown of the vortices generated from the foil's surface. Two methods are employed to visualize the vortical patterns of the moving/deforming foils: vorticity criterion and *Q* criterion [52]. Vorticity criterion uses the values of vorticity computed by

(7) $$\begin{array}{c} \vec{\omega }=\vec{\nabla }\times \vec{V}. \end{array}$$

The *Q* criterion is based on the value of the second invariant of the velocity gradient tensor $\nabla \vec{v}$ [52] as follows:

(8) $$\begin{array}{c} Q=\Omega_{ij}\Omega_{ij}-S_{ij}S_{ij}, \end{array}$$

where *Ω*_*ij*_ and *S*_*ij*_ are the antisymmetric and symmetric components of $\nabla \vec{v}$, respectively, which are defined as follows:

(9) Ωij12∂vi∂xj−∂vj∂xi,Sij=12∂vi∂xj+∂vj∂xi.

Physically, *Q* can be a balance between the strain rates ($S=\sqrt{S_{ij}S_{ij}}$) and the rotation ($\Omega =\sqrt{\Omega_{ij}\Omega_{ij}}$). Thus, positive values of *Q* indicate regions where the strength of rotation (rotation rate) dominates the strength of strain (strain rate) and hence, *Q*-isosurfaces can denote the vortex envelopes [52].

### 2.10. Forces, Consumed Power, and Efficiency Calculations

Momentum transfer of the foil to the surrounding water and vice versa is via drag, lift, and thrust [40] which are produced from the pressure and velocity distributions (provided from the fluid solver) on foil's body due to the flapping and undulating motions. The foil has unsteady or time dependent periodic lateral movements and can also have unsteady or steady inline motions. Inline unsteady motions are known as accelerating or braking. For the steady propulsion, the forces and moments acting on the foil are balanced. The pressure forces are calculated by integration of the pressure distribution (which is normal to the surface) over the foil's surface. For an incompressible flow over an impenetrable surface, the normal stress force is zero and integration of only the viscous shear stresses over surface results in the viscous forces. The integrations are as follows:

(10) Fvi∫ΓτijnjdS,i≠j,Fpi=∫ΓpnidS,Fi=Fpi+Fvi,

where *F*_*v*_ and *F*_*p*_ are the viscous and pressure forces, respectively, Γ is foil's surface, *τ*_*ij*_ (*i* ≠ *j*) are tangential components of the stress tensor on the surface, *n* is the unit normal vector on the surface element (*dS*), *p* is the pressure value on the foil. Thrust and drag forces are the sum of all forces in the direction or the counter direction of motion, respectively.

The propulsion performance of an oscillating airfoil is represented with three important parameters: mean input power ($\overset{-}{P}$), mean inline force (${\overset{-}{F}}_{X}$), and propulsion efficiency (*η*). The mean input power is expressed as

(11) P¯1T∫0TP dt=1T∫0TFydhtdtdt+∫0TMtdθdtdt,

where *dh*(*t*)/*dt* and *F*_*y*_(*t*) are the transvers displacement rate and the force component in the direction of *y*-axis, respectively. *dθ*/*dt* and *M*(*t*) are rates of changes in the pitching angle and the value of pitching moment in *xy* plane, respectively. Finally, the overall propulsion efficiency is calculated by

(12) $$\begin{array}{c} \eta =\frac{{\overset{-}{F}}_{X}U_{\text{ref}}}{\overset{-}{P}}. \end{array}$$

## 3. Validation, Grid Dependency Study, and Time Step

To verify the accuracy, convergence, and stability of the solver, a series of validation tests are performed along with the grid dependency tests. Also, the value of time step for each simulation is selected in such a way that the solver can capture the smallest grid size with respect to the inlet and moving grid velocities.

### 3.1. Validation of Solver

A high-aspect-ratio foil, illustrated at Figure 13, with chord length *c*, moves at constant forward speed *U*, performing a heaving motion, *h*(*t*), of amplitude *h*_0_ and frequency *ω*, and a pitching motion, *θ*(*t*), of amplitude *θ*_0_ and frequency *ω*. The pitching motion has a phase lead with respect to the heaving motion, which is denoted by *ψ*. The one-third-chord point is the pivot point. The true angle of attack profile can be calculated mathematically as follows:

(13) αtarctan⁡h˙tUref−θt=arctan⁡πStcos⁡⁡ωt−θ0sin⁡ωt+ψ,St2h0fU.

The foil oscillates in a flapping motion around one-third chord with the heaving amplitude of 0.75*c* (*c* = 0.1 m). Two cases are used for the validation purposes.

**Case 1 .**

There are different maximum angles of attack, *α*_0_ = {5°, 10°, 15°, 20°, 25°, 30°}, for a selected Strouhal number St = 0.2 (Figure 14(a)).

**Case 2 .**

There are different Strouhal numbers, St = {0.1,0.2,0.3,0.4,0.5,0.6}, for a selected maximum angle of attack *α*_0_ = 15° (Figure 14(b)).

The maximum pitch angle for *ψ* = 90° can be determined approximately as follows [30]:

(14) $$\begin{array}{c} \theta_{0}=\arctan\left(\;\pi \text{St}\;\right)-\alpha_{0}. \end{array}$$

In Case 1, the maximum angle of attack varies in the range of [5°, 30°] at a constant Strouhal number St = 0.2 and a constant phase difference *ψ* = 90°. Reynolds number is set to be 40000. Figures 15(a)–15(c), respectively, show the results of thrust and power coefficients and efficiency for the solver with medium grid size of 20436 nodes and the comparison with the analytical and experimental results of Anderson et al. [30] and numerical results of Xiao and Liao [53].

In Case 2, the Strouhal number varies in the range of [0.1, 0.6] at a constant maximum angle of attack *α*_0_ = 15° and a constant phase difference *ψ* = 90°. Reynolds number is also set to be 40000. Figures 16(a)–16(c), respectively, show the results of thrust and power coefficients and efficiency for the solver with medium grid size of 20436 nodes and the comparison with the analytical and experimental results of Anderson et al. [30] and numerical results of Xiao and Liao [53].

### 3.2. Grid Dependency

Figures 17 and 18 summarize the results for the grid dependence study. Here the results are presented for the case of *Re* = 40000 using three grid sizes of 10124, 20436, and 40732 nodes. All tests of Cases 1 and 2 have repeated to evaluate the dependence of results on domain resolution. Based on this grid dependence study, it is concluded that the medium grid size of 20436 is sufficient for carrying out the simulations which are insensitive to grid size.

## 4. Results and Discussions

**Figure 4:**
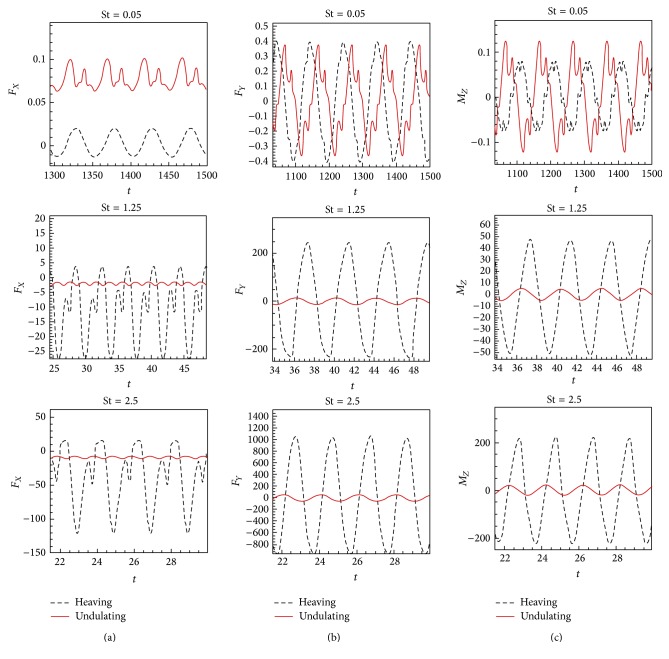
Time histories of (a) the inline force, (b) the transverse force, and (c) the moment of the heaving and undulatory oscillations at three Strouhal numbers St = 0.05, St = 1.25, and St = 2.5, respectively, in *Re* = 40000.

**Figure 5:**
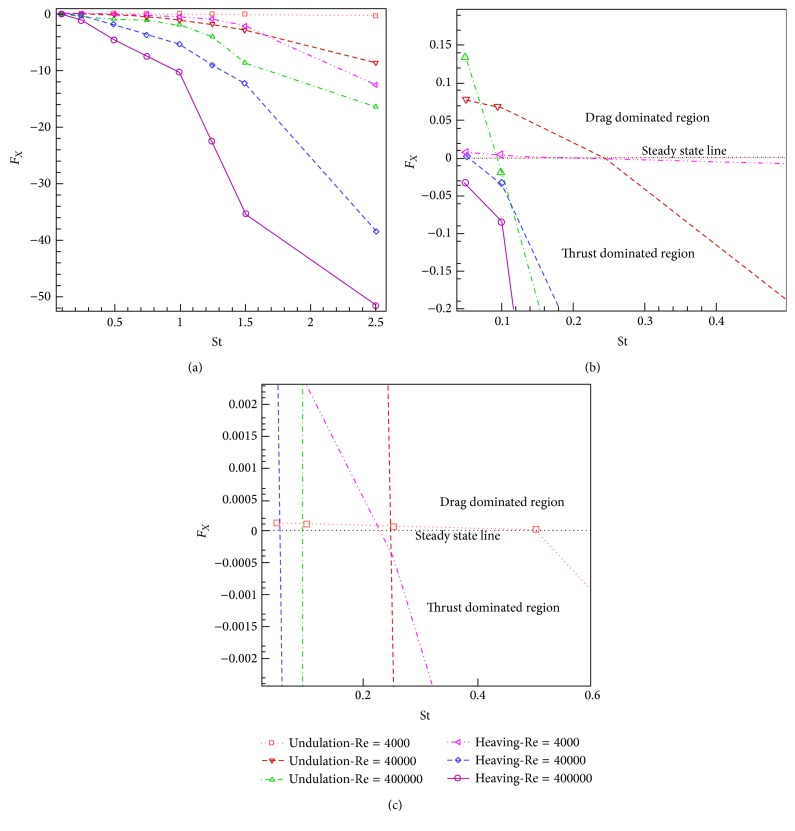
The time averaged inline force of heaving and undulatory oscillations versus Strouhal number. (a) Full range of Strouhal numbers, and (b, c) zoomed area to show the steady state line.

**Figure 6:**
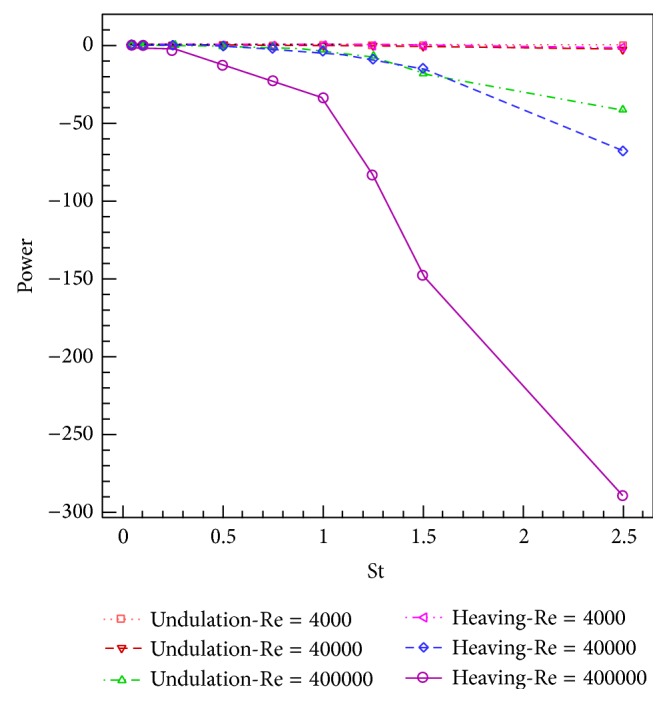
The time averaged consumed power for heaving and undulatory oscillations versus Strouhal number.

**Figure 7:**
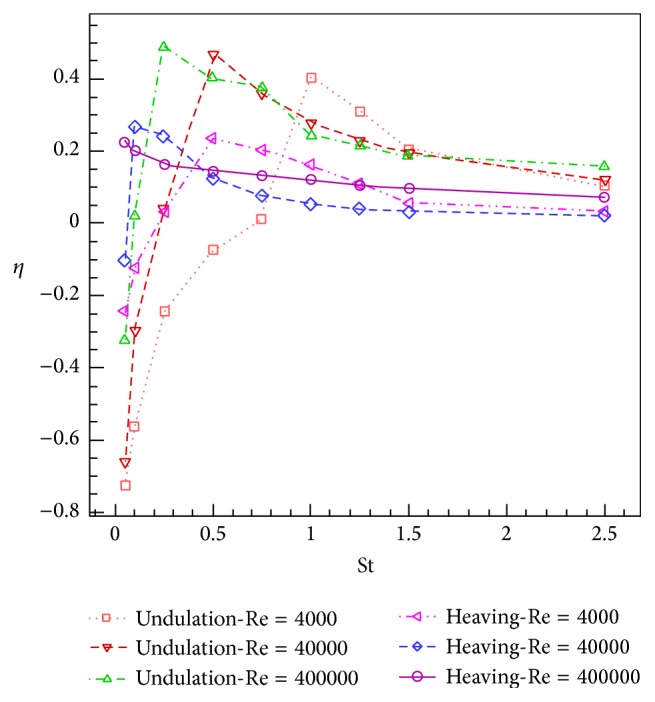
Efficiency of heaving and undulatory oscillations versus Strouhal number.

**Figure 8:**
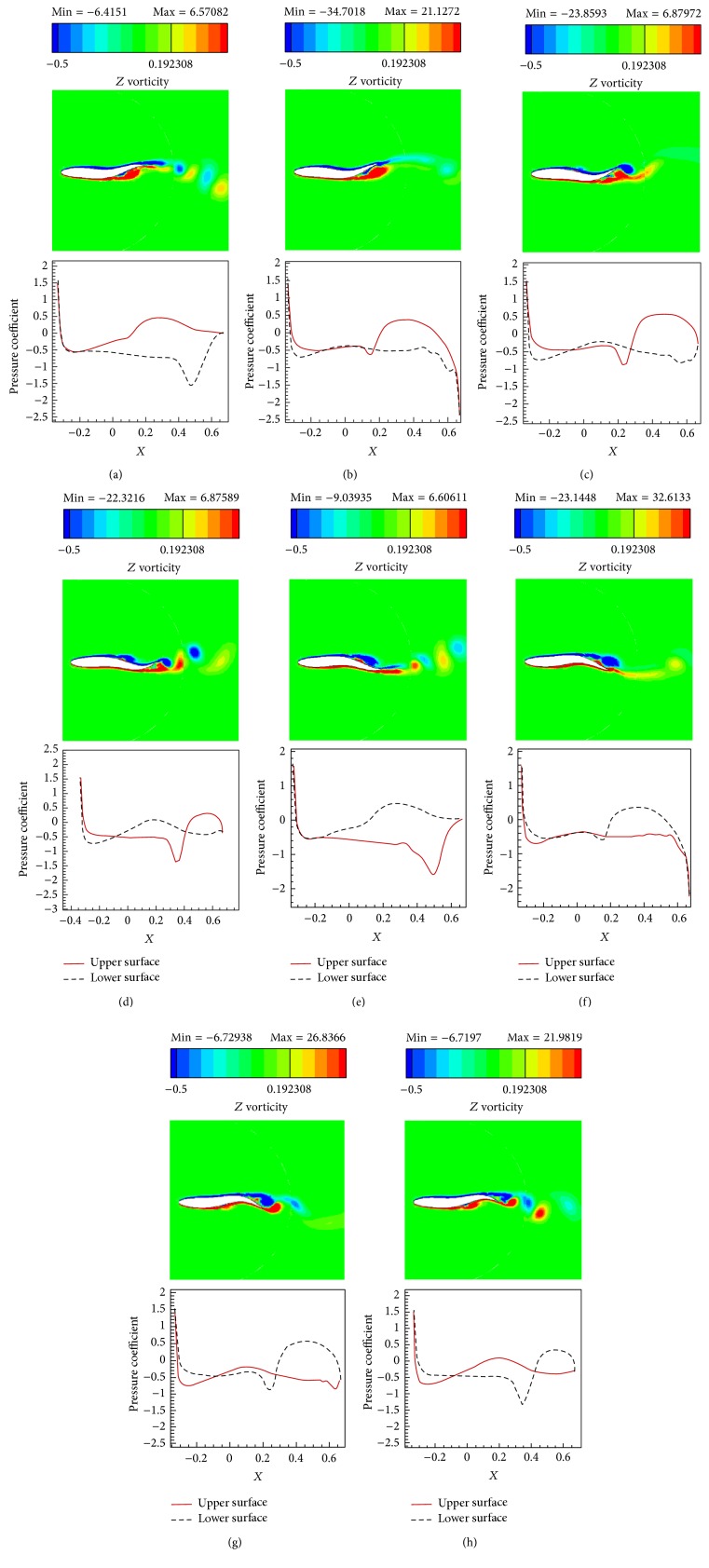
Vorticity contours and foil's surface pressure distribution at eight time instants within one period of oscillations for the undulating foil at St = 0.05, *Re* = 40000. (a) *t* = *t*_0_, (b) *t* = *t*_0_ + *T*/8, (c) *t* = *t*_0_ + 2*T*/8, (d) *t* = *t*_0_ + 3*T*/8, (e) *t* = *t*_0_ + 4*T*/8, (f) *t* = *t*_0_ + 5*T*/8, (g) *t* = *t*_0_ + 6*T*/8, and (h) *t* = *t*_0_ + 7*T*/8.

**Figure 9:**
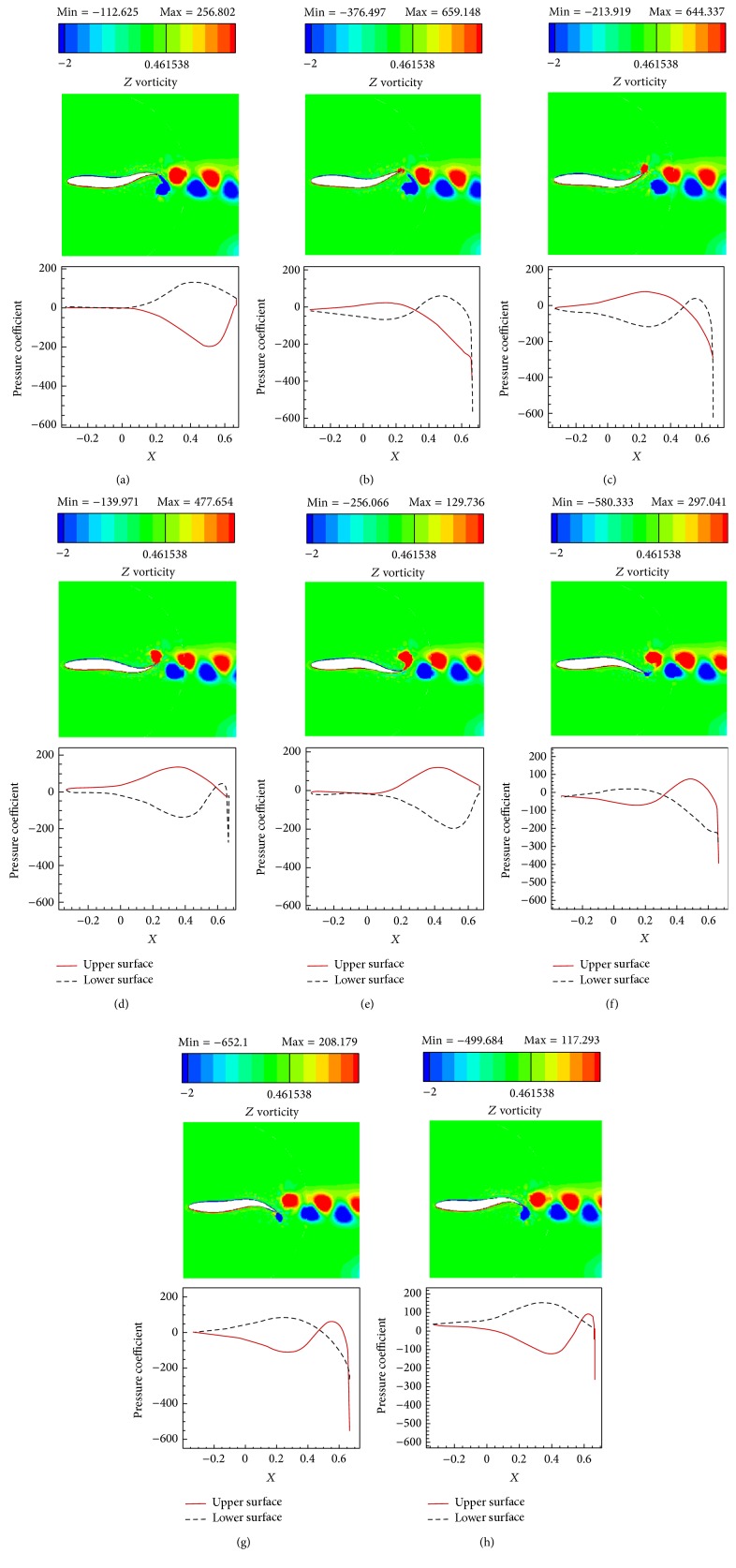
Vorticity contours and foil's surface pressure distribution at eight time instants within one period of oscillations for the undulating at St = 2.5, *Re* = 40000. (a) *t* = *t*_0_, (b) *t* = *t*_0_ + *T*/8, (c) *t* = *t*_0_ + 2*T*/8, (d) *t* = *t*_0_ + 3*T*/8, (e) *t* = *t*_0_ + 4*T*/8, (f) *t* = *t*_0_ + 5*T*/8, (g) *t* = *t*_0_ + 6*T*/8, and (h) *t* = *t*_0_ + 7*T*/8.

**Figure 10:**
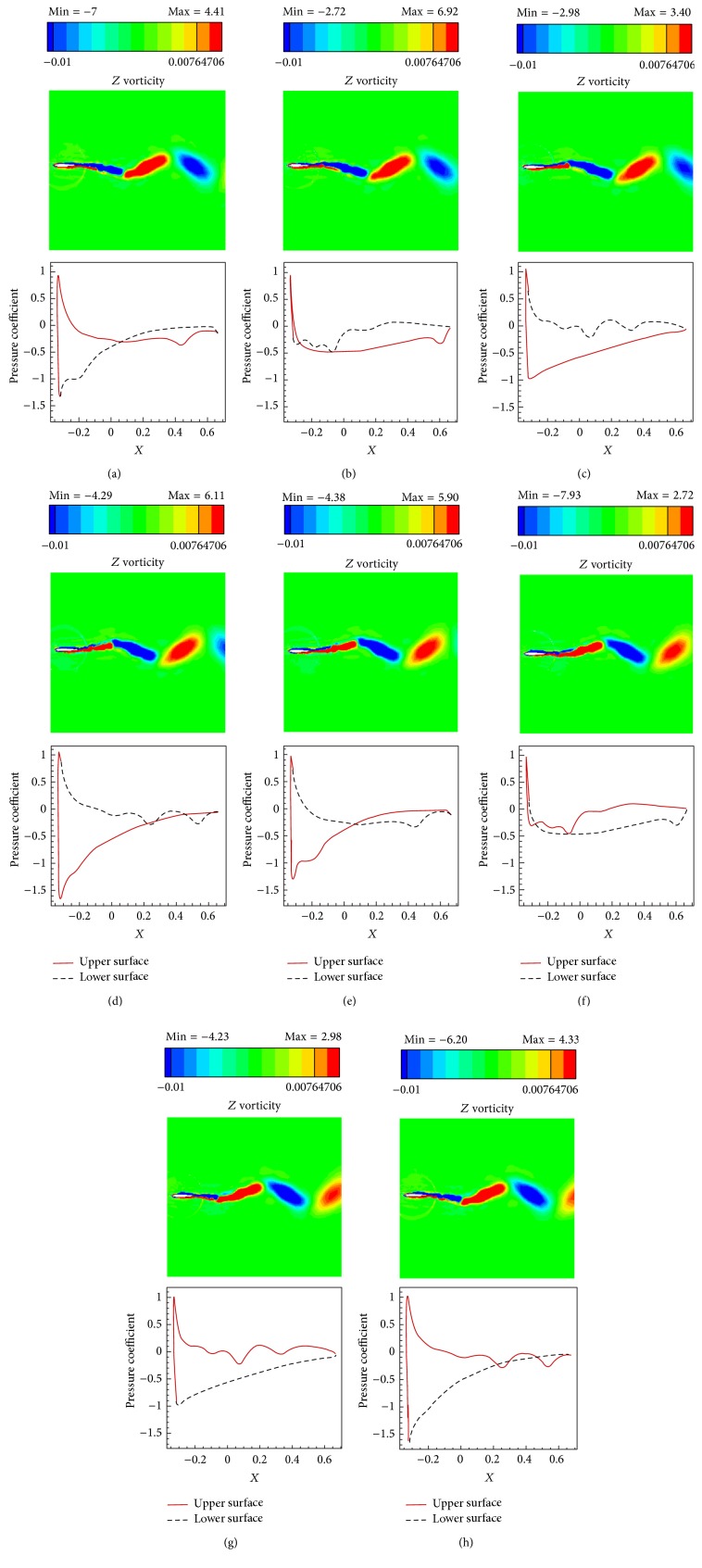
Vorticity contours and foil's surface pressure distribution at eight time instants within one period of oscillations for the heaving foil at St = 0.05, *Re* = 40000. (a) *t* = *t*_0_, (b) *t* = *t*_0_ + *T*/8, (c) *t* = *t*_0_ + 2*T*/8, (d) *t* = *t*_0_ + 3*T*/8, (e) *t* = *t*_0_ + 4*T*/8, (f) *t* = *t*_0_ + 5*T*/8, (g) *t* = *t*_0_ + 6*T*/8, and (h) *t* = *t*_0_ + 7*T*/8.

**Figure 11:**
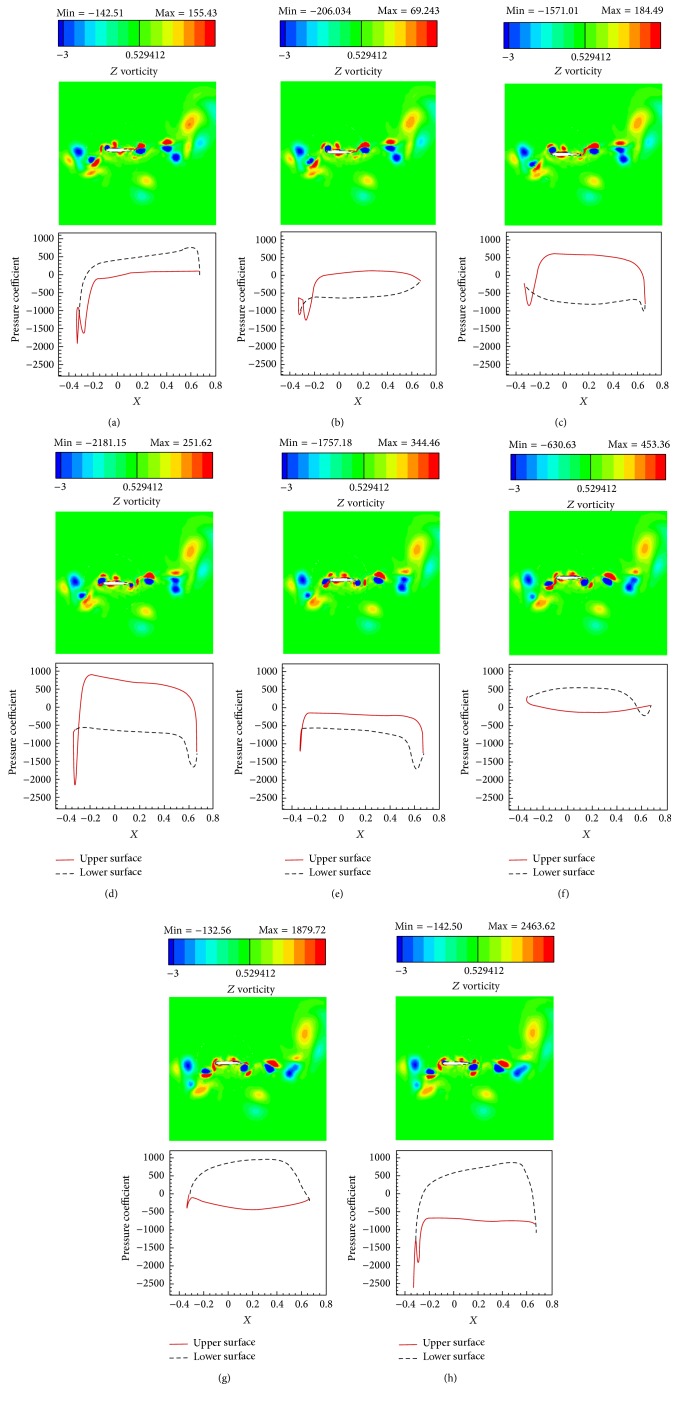
Vorticity contours and foil's surface pressure distribution at eight time instants within one period of oscillations for the heaving foil at St = 2.5, *Re* = 40000. (a) *t* = *t*_0_, (b) *t* = *t*_0_ + *T*/8, (c) *t* = *t*_0_ + 2*T*/8, (d) *t* = *t*_0_ + 3*T*/8, (e) *t* = *t*_0_ + 4*T*/8, (f) *t* = *t*_0_ + 5*T*/8, (g) *t* = *t*_0_ + 6*T*/8, and (h) *t* = *t*_0_ + 7*T*/8.

**Figure 12:**
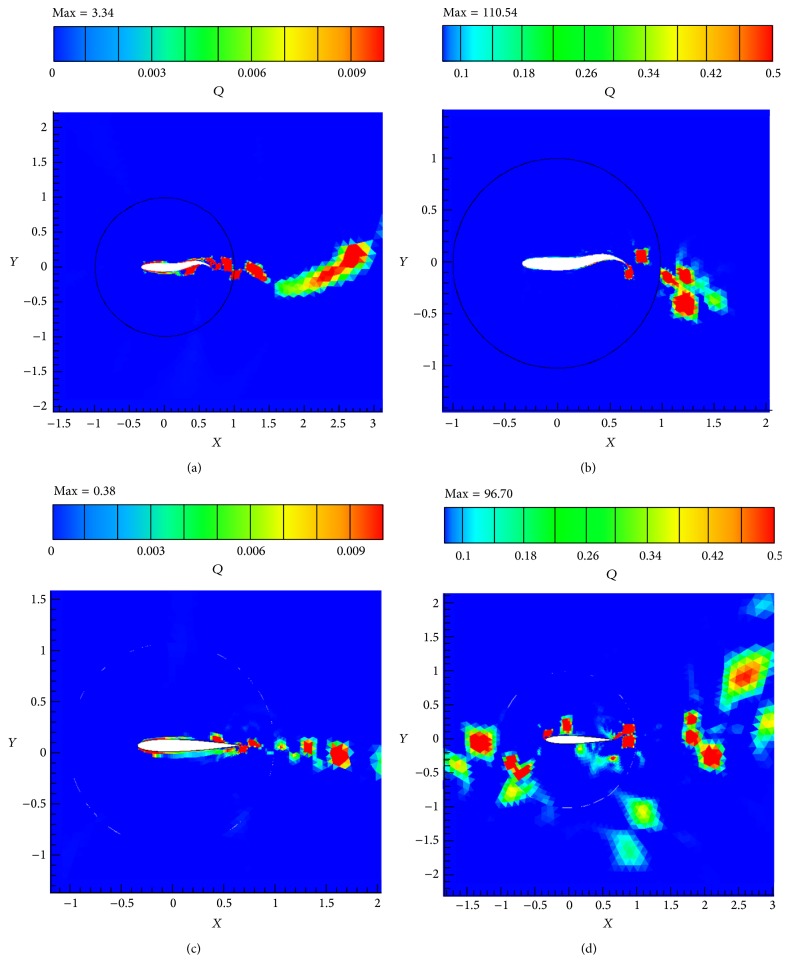
The *Q* criterion values showing the footprints of the wake structure for (a) the undulating foil at St = 0.05, (b) the undulating foil at St = 2.5, (c) the heaving foil at St = 0.05, and (d) the heaving foil at St = 2.5, in *Re* = 40000.

**Figure 13:**
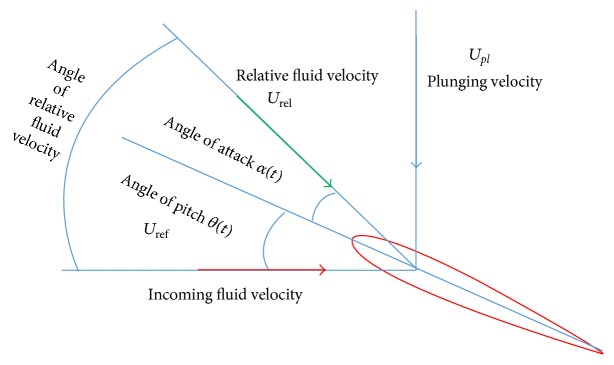
Vector diagram for velocity components relative to heaving and pitching motions of foil and incoming fluid velocity.

**Figure 14:**
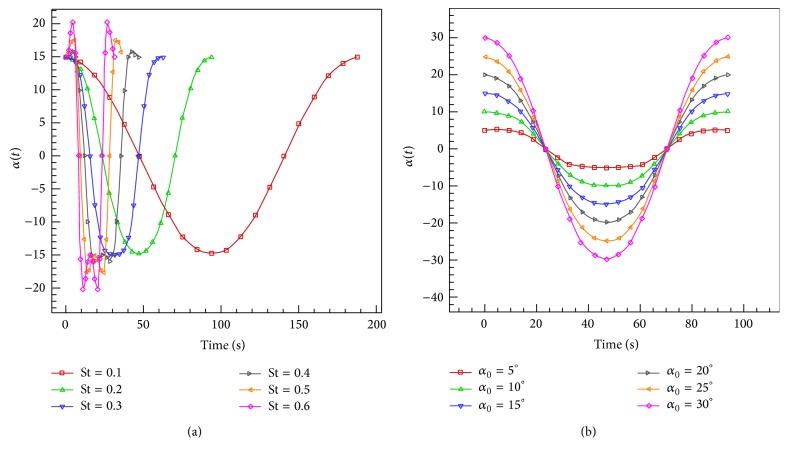
Variation of effective angle of attack *α*(*t*) profile versus (a) maximum angle of attack *α*_0_ at constant St = 0.2 and (b) St at constant *α*_0_ = 15°.

**Figure 15:**
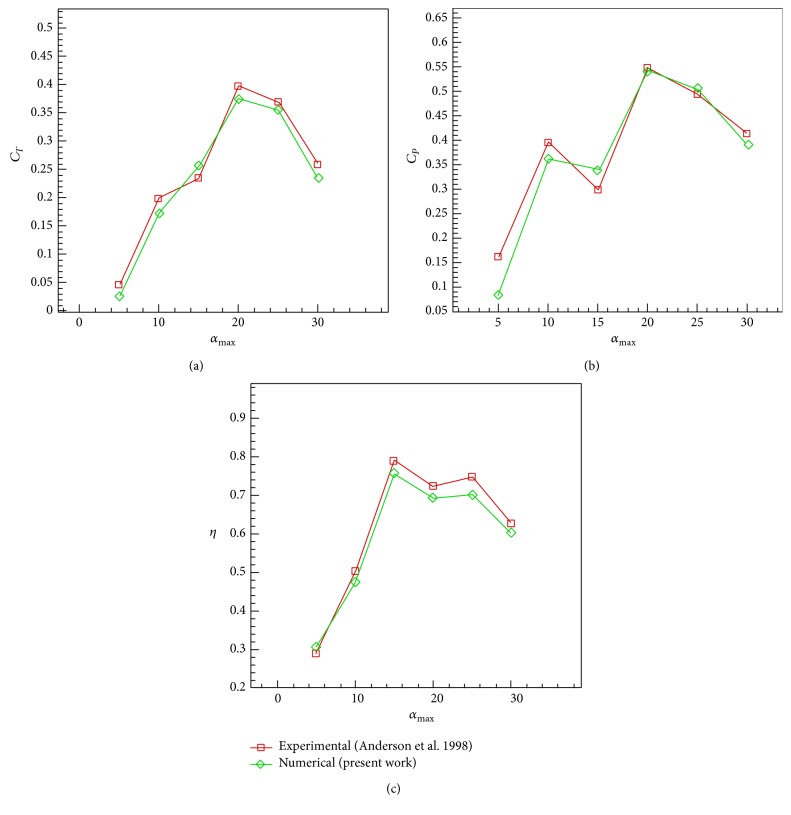
Comparison of simulation results with the experimental results of Anderson et al. [30] with *h*_0_ = 0.75, *ψ* = 90°, and St = 0.2 versus maximum angle of attack. (a) Mean thrust coefficient. (b) Mean power coefficient. (c) Efficiency.

**Figure 16:**
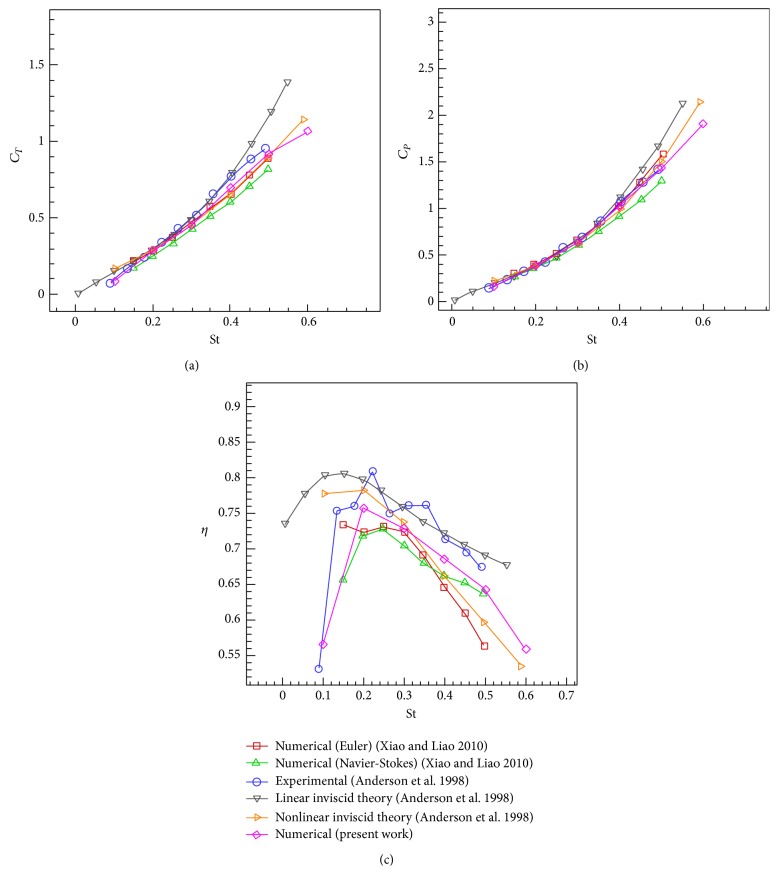
Comparison of simulation results with the analytical and experimental results of Anderson et al. [30] and numerical results of Xiao and Liao [53] with *h*_0_ = 0.75, *ψ* = 90°, and *α*_0_ = 15° versus Strouhal number. (a) Mean thrust coefficient. (b) Mean power coefficient. (c) Efficiency.

**Figure 17:**
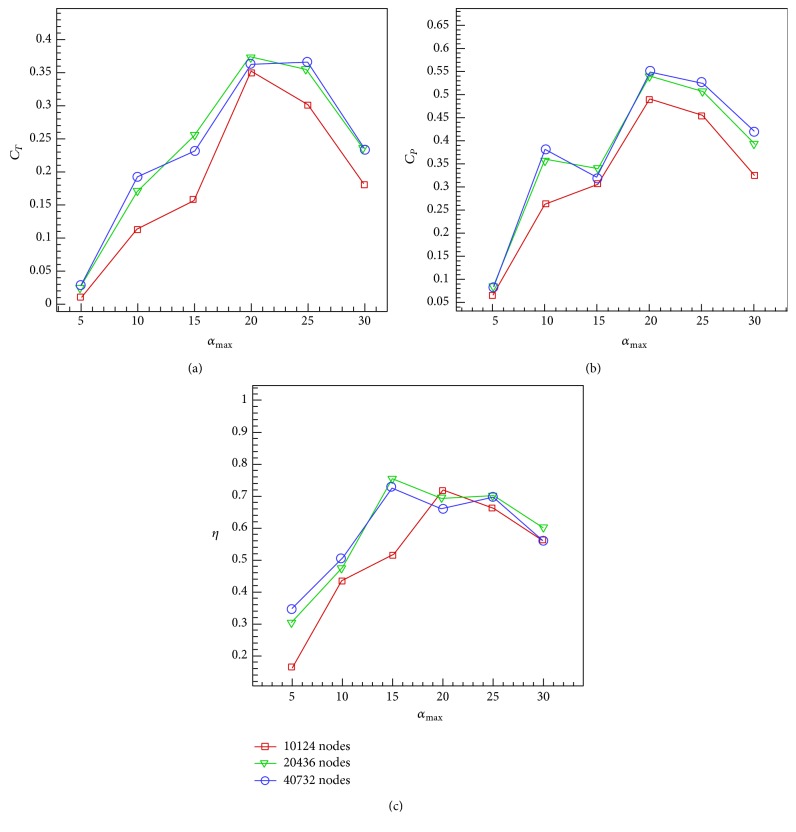
Comparison of simulation results of three grid resolutions of fine (10124 nodes), medium (20436 nodes), and course (40732 nodes) meshes for *h*_0_ = 0.75, *ψ* = 90°, and St = 0.2 versus maximum angle of attack. (a) Mean thrust coefficient. (b) Mean power coefficient. (c) Efficiency.

**Figure 18:**
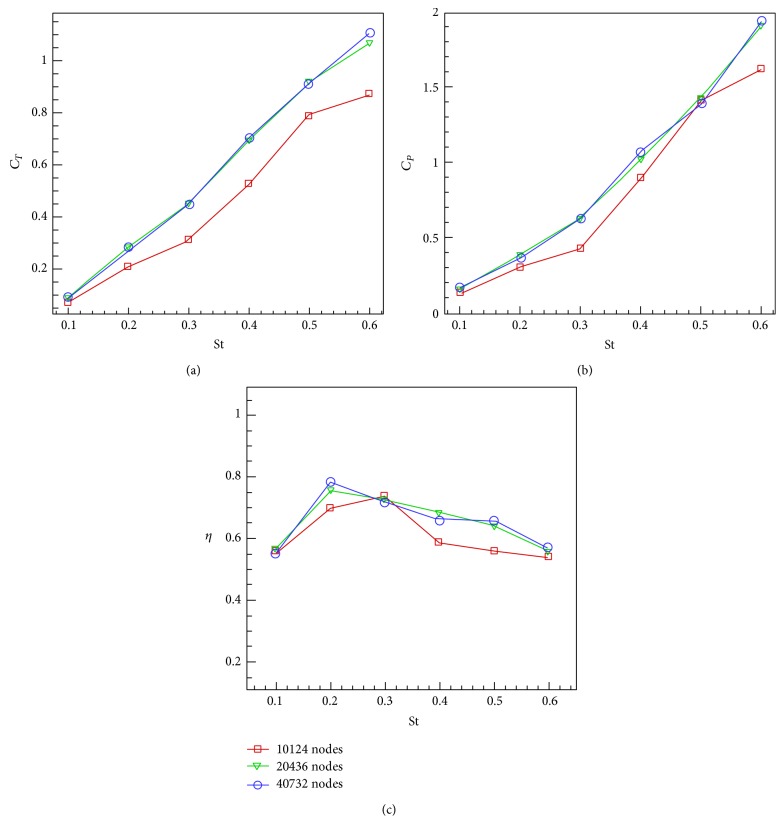
Comparison of simulation results of three grid resolutions of fine (10124 nodes), medium (20436 nodes), and course (40732 nodes) meshes for *h*_0_ = 0.75, *ψ* = 90°, and *α*_0_ = 15° versus Strouhal number. (a) Mean thrust coefficient. (b) Mean power coefficient. (c) Efficiency.

The simulations are carried out for the cases of heaving and undulatory oscillations at *Re* = 4000, 40000, and 400000, a maximum trailing edge peak to peak amplitude of 0.2*L* (*L* is the chord length of the foil), and the Strouhal numbers in the range of [0.05, 2.5]. The solutions are continued to multiple periods of oscillations to ensure the repetitive periodic time dependent results (as seen in Figure 4). This systematical investigation reveals the comparative behaviors of the energetics of the heaving and undulatory oscillations as well as their flow patterns. Comparisons of computational results cover the predictions of instantaneous inline and transverse forces and 12 moment (Figure 4), time-averaged inline force (Figure 5), consumed power (Figure 6), and efficiency (Figure 7), versus St, as well as vorticity (Figures 8–11) and *Q* criterion (Figure 12) visualizations of the flow patterns at eight time instants of a period of oscillations for low and high Strouhal numbers. The maximum and minimum values of vorticity and the maximum value of *Q* (*Q* > 0 is meaningful for vortical pattern) are presented on the top of each plot. In addition, the surface pressure coefficients of the undulating and heaving foils are included in Figures 8–11 at the corresponding time instants.

### 4.1. Instantaneous Inline and Transverse Forces and Pitching Moment

The instantaneous inline and transverse hydrodynamic forces and pitching moment, which are shown in Figure 4, are presented to bring out qualitative insights of the differences in time varying results at different Strouhal numbers. The foils are assumed to be restrained by rigid bases, where the net hydrodynamic forces and the pitching moment are absorbed by these hypothetical bases. Therefore, the inline forces, shown in Figure 4(a), are net forces that would be available to accelerate the foil either forward or backward, depending on the sign of the mean time value, when the hypothetical bases are removed. The forward or positive acceleration occurs when the propulsive or thrust force exceeds the resistive or drag force. In contrast, when the drag force dominates the thrust force, the negative or backward acceleration occurs.

From Figure 4, it can be found that, to reach a certain value of inline force (thrust), much energy is lost due to oscillations of the traverse and opposing inline forces and moment in each cycle. Since all the existing thrust producing mechanisms suffer from the energy losses, the more efficient mechanism among them should be selected. The energy losses mostly originate from the vortical structures of oscillating mechanisms. The vortical structure is created due to interactions of the foil body and its surrounding fluid. Hence, the differences between the oscillation mechanisms would originate from the differences of their wake structures which inherently are due to their different kinematics and rigidity/flexibility.

The approximate ratios of the peak amplitudes of oscillations of the instantaneous inline and transverse forces and the moment of the heaving mechanism over the undulating mechanism are, respectively, 0.9, 1.1, and 0.6 at St = 0.05, respectively, 16, 10, and 10 at St = 1.25, and, respectively, 28, 21, and 11 at St = 2.5, in *Re* = 40000. These values show that the energy losses due to the lateral oscillations of both mechanisms are approximately in the same order at low St (St = 0.05) and as could be seen from Figures 8 and 10, the low St wakes of heaving and undulating foils show similar patterns. However, the ratios are about one order greater at medium St (St = 1.25) and also at high St (2.5) are approximately twice the medium St. In other words, the energy losses of the heaving foil due to the interactions with the surrounding fluid are much more than the undulating foil especially at medium and high St. It is confirmed by looking at Figures 9 and 11, where the wake structures of heaving and undulating foils are very different at high St.

### 4.2. Time Averaged Inline Forces (Thrust)

The time averaged inline forces (Figure 5) show the transition from the drag dominated regime to the thrust dominated regime. In other words, the Strouhal number of the oscillations determines the drag dominated, thrust dominated, and neutral propulsion states. At low St, the oscillations are drag dominated. Lower frequency of oscillations produces lower energy transmissions from the foil body to the surrounding fluid and hence, the generated thrust cannot dominate the resistive forces. On the other hand, at higher St, due to higher energy transmissions, the propulsive forces can dominate the resistive forces. The neutral Strouhal numbers of heaving St_*H*_^∗^ and St_*U*_^∗^ undulations, in which the transitions from drag dominated regime to thrust dominated regime occur, are St_*H*_^∗^ = 0.23 and St_*U*_^∗^ = 0.5 in *Re* = 4000, St_*H*_^∗^ = 0.05 and St_*U*_^∗^ = 0.25 in *Re* = 40000, and St_*H*_^∗^ < 0.01 and St_*U*_^∗^ = 0.09 in *Re* = 400000 (Figures 5(b) and 5(c)). At neutral Strouhal number or “steady state Strouhal number,” the propulsor produces the thrust force same as the existing drag force. Therefore, there is not any acceleration and hence, the propulsor moves with constant speed.

Although, at low St, below the steady state St, the inline force shows some positive values, it descends monotonously, due to the direction of the *x*-axis, as St is increased. However, as shown in Figure 5(a), the curves corresponding to the undulating foil descend with lower slopes than the heaving foil. The differences of descending slopes show that, in given Reynolds and Strouhal numbers, heaving motions produce higher thrust. In particular, at higher St, the produced thrust by heaving foil is much more than the undulating foil in a given *Re*. It means the undulating mechanism has some limitations to produce high thrust forces. However, considering solely the amount of thrust production is a single attitude to the performance. Thus, for a comprehensive evaluation of the performance of these mechanisms, other parameters such as power consumption and efficiency should also be considered.

Comparing the curves shown in Figure 5, increasing the Reynolds number of flow from 40000 to 400000 causes the frequency of undulating and heaving motions to be increased in order to make the Strouhal number remain constant. Hence, as it is seen, the time averaged inline forces are increased due to increasing the *Re*. In contrast, by decreasing the *Re* and keeping the St constant, the produced inline forces are also decreased. In addition, the change of *Re* causes the movement of the steady state Strouhal numbers St_*H*_^∗^ and St_*U*_^∗^, forward or backward, respectively, by decreasing or increasing *Re*.

### 4.3. Consumed Power

The consumed power (Figure 6) also descends monotonously with increasing the St, due to the direction of the *x*-axis. However, the meaning of single sign of the power is that, to oscillate a foil, always some power must be consumed. As Figure 6 indicates, the power curve of the undulating foil descends with a lower slope than the heaving foil. In other words, in given St and *Re*, especially at higher St, the heaving mechanism needs much more power than the undulating mechanism. However, at low St (approximately St ≤ 0.3), nearly same powers are required to oscillate the foils with both undulating and heaving kinematics. It is also confirmed by looking at the similar vortical patterns shown in Figures 8 and 11. Furthermore, by comparison of the rates of changes in the power curves with inline force curves, in a given *Re*, it could be found that the difference between the descending slopes of power curves grows more intensely. The significances of power consumption and energy costs are highlighted in the autonomous underwater vehicles, because of limited availabilities to energy in their operational domains and also their limiting design conditions such as weight. In addition, increasing the *Re* from 4000 to 40000 and keeping the St constant causes increase of the power consumption of the oscillating foils with great differences in higher *Re*.

### 4.4. Efficiency

Figure 7 indicates that the efficiency curves show remarkable behaviors with respect to the Strouhal number. At low St, because of opposite signs of the output power and the consumed power, the values of efficiency are obtained negative. With increasing the St, the efficiency reaches positive value called “peak efficiency point,” St_*H*_^PE^ and St_*U*_^PE^ for the heaving and undulating foils, respectively. In the peak efficiency point, maximum thrust is produced with minimum power consumptions. The peak efficiency points occur at St_*H*_^PE^ = 0.5 and St_*U*_^PE^ = 1.0 in *Re* = 4000, St_*H*_^PE^ = 0.05 and St_*U*_^PE^ = 0.5 in *Re* = 40000, and St_*H*_^PE^ = 0.01 and St_*H*_^PE^ = 0.25 in *Re* = 400000. The value of the peak efficiency point is called “peak propulsive efficiency,” *η*_*H*_^*P*^ and *η*_*U*_^*P*^ for the heaving and undulating foils, respectively. The peak propulsive efficiency values are *η*_*H*_^*P*^ = 0.24 and *η*_*U*_^*P*^ = 0.4 in *Re* = 4000, *η*_*H*_^*P*^ = 0.27 and *η*_*U*_^*P*^ = 0.47 in *Re* = 40000, and *η*_*H*_^*P*^ = 0.22 and *η*_*U*_^*P*^ = 0.49 in *Re* = 400000. After the peak efficiency point, at higher St, the value of efficiency decreases monotonously from the peak propulsive efficiency. The ascending and descending behaviors of the efficiency curves, from lower to higher Strouhal numbers, can be attributed to the vortical patterns of the oscillation mechanisms. However, the focus on these features requires scrutinized investigations of the vortical patterns and also the evolution of the vortices from forming to decaying stages at different Strouhal numbers.

A comparison of the efficiency curves of heaving and undulatory mechanisms in a given *Re* brings out the fact that, at low Strouhal numbers, where the efficiency is less than the peak efficiency point of the undulating foil, the value of efficiency for heaving oscillations is greater than the efficiency of the undulating oscillations. After the peak efficiency point of the heaving foil, at higher Strouhal numbers, the efficiency of undulatory oscillations reaches higher values than heaving oscillations. The difference between the efficiency values of heaving and undulating foils is greater at regions near the peak efficiency point of undulating foil, while this difference decreases by receding from that point. Mostly higher efficiency behavior of undulation mechanism, comparing with the heaving mechanism, shows a good potential of applying this mechanism to design of the underwater vehicles and the stabilizing control surfaces.

By comparing the efficiency curves in different *Re*, it is obvious that changes in *Re* move the peak efficiency points and alters the efficiency values. For undulating motion, increasing the *Re* from 4000 to 400000 increases *η*_*U*_^*P*^. But this behavior does not occur for heaving motion, since the maximum *η*_*H*_^*P*^ is for *Re* = 40000 and the minimum *η*_*H*_^*P*^ is for *Re* = 4000.

### 4.5. Wake Structures

The wake of oscillating foils has already been studied experimentally using Particle Image Velocimetry (PIV) and numerically using the CFD simulations. These studies have showed the generation of vortices in the downstream wake and few works have considered comparatively the vortical patterns of different oscillation mechanisms. This paper does not aim to deeply investigate the wake structures of undulating and heaving mechanisms, but some important comparative and qualitative features at low and high Strouhal numbers are presented to recognize how significant discrepancies between the performance and the effects of these oscillation mechanisms might originate from their vortical patterns. However, to discover the wake phenomenon and its nature and also to distinguish in detail the qualitative and quantitative discrepancies of wake of these mechanisms, more deep researches are required to focus on the wake structure, the vortex-body or vortex-vortex interactions, and the evolution of the vortices from formation to decay.

From low St vorticity contours (Figures 8 and 10) and *Q* values (Figures 12(a) and 12(c)), some similarities especially at trailing edge region can be observed in the vortical patterns of heaving and undulating mechanisms. This is attributed to the weakness of the vortices. In contrast, there are many discrepancies between their vortical patterns at high St (Figures 9, 11, 12(b), and 12(d)). Furthermore, Figures 8 and 10 show that, in the vicinity of trailing edge, distinct vortices cannot be observed at low St. On the other hand, these distinct vortices at low St are formed in a specific time instance and also at especial distance from the trailing edge. However, the delay on the formation of distinct vortices in the heaving foil is greater than the undulating foil. It is probably due to the stronger vortices in the undulating foil's wake (as denoted by maximum and minimum values on the top of each plot). The generated jets behind the foils and hence the produced thrust from the pairs of counter rotating vortices are also weak at low St. Furthermore, the vortices disappear in a short length behind the trailing edge because of the effects of the viscosity of medium.

The wake pattern of both oscillatory mechanisms depends on the operating Strouhal number. At higher Strouhal numbers, stronger vortices are produced and they are shed into the downstream at the tip of the trailing edge. The produced vortices in the leading and trailing edges of the heaving foil surface are both strong at higher Strouhal numbers. From Figures 9 and 11, it could be inferred that the produced jets behind the foils from the pairs of counter rotating vortices are strong due to the strong vortices. The produced vortices at higher St disappear in further distances behind the trailing edge in comparison to the lower St. The most significant difference between the vortical patterns of the heaving and undulating foils is the production of leading edge vortices by heaving oscillations. This feature can considerably affect the power consumption and the efficiency of the oscillation mechanisms (Figures 6 and 7). It is worth to note that, in some applications, the vortical signature and trace of propulsion or stabilizing mechanisms are particularly significant.

As mentioned, the positive values of *Q* indicate regions where the strength of rotation (rotation rate) dominates the strength of strain (strain rate) and hence, *Q*-isosurfaces could denote the vortex envelopes. For example, the *Q* criterions shown in Figure 12 confirm our findings about the vortical patterns at low and high Strouhal numbers. In addition, better quantitative comparisons could be achieved from the *Q* criterion. From the values indicated at top of Figure 12, the ratios of maximum *Q* values of undulating over heaving oscillations are approximately 8.79 and 1.14, at low and high St, respectively. In addition, the ratios of low over high St maximum values of *Q* for undulating and heaving oscillations are approximately 33.1 and 254.47, at low and high St, respectively. It might be suggested that the lower power consumption and higher efficiency behaviors of undulating oscillations at higher Strouhal numbers are directly related to its vortical structure and the strengths of the formed vortices as well as their arrangements. Furthermore, as mentioned by vorticity criterion, the *Q* pattern of undulating foil shows more regular pattern. The primary reason is the appearance of the leading edge vortices for the heaving foil and their interactions with each other and/or with downstream vortices.

Studies of the unsteady hydrodynamics of oscillating motions of flexible and rigid fins have suggested that a rich set of phenomena exist, depending on the nondimensional frequency of oscillations, the wavelength of the excitation, and the aspect ratio of the fin. In some cases, [54], Zhang et al. [26], and also this study, simple wake structures have been observed that bear a strong resemblance to the structure of coflowing jets and wakes. In other cases, such as the work of Moored et al. [55], Dewey et al. [56], and Borazjani and Sotiropoulos [57] bifurcating wakes are seen, and both cases appear to correspond to a peak in efficiency. Moored et al. [55] developed an alternative framework, based on hydrodynamic wake. They found that local optima in propulsive efficiency occur when the driving frequency of a flapping fin matches the hydrodynamic resonant frequency of the jet profile and there can be multiple wake resonant frequencies and modes corresponding to multiple peaks in efficiency resonance theory [55]. However, in our cases, due to the geometry and kinematic parameters, the single row of vortices [57] called* 2S* wake pattern [55] with a peak efficiency point for each mechanism is observed.

### 4.6. Surface Pressure Coefficients

The pressure coefficients along foils surface, illustrated in Figures 8 to 11, can be separately discussed for lower and upper Strouhal numbers. At low Strouhal numbers, due to the weakness of the oscillation motions, fluctuations of pressure are also small. Maximum and minimum pressure coefficients at St = 0.05 are in the orders of 1 for both heaving and undulating foils. Furthermore, at low St, the undulating foil shows special behaviors in pressure coefficient graphs with jumps at its upper and lower surfaces. However, the value of pressure coefficient of the heaving foil varies more smoothly than the undulating foil. The most significant reason of these jumps could be attributed to the formation of vortices at the foil surface, because of the deformations of the posterior part of the undulating foil. On the other hand, the formation of vortices for the heaving foil begins at far downstream of the foil at low St. In contrast, at high St, due to the strong oscillations, fluctuations of pressure are also large. For example, the absolute values of the maximum and minimum pressure coefficients at St = 2.5 are in the orders of 100 and 1000 for undulating and heaving motions, respectively. Although the jumps of the surface pressure coefficients can be observed for both heaving and undulating foils at high St, the positions of jumps are different. The heaving oscillations show both leading and trailing regions' jumps as well as high values of pressure coefficient in the lateral regions due to the corresponding vortices, while undulating oscillations just indicate trailing region's jumps.

## 5. Conclusions

In the present study, the simulations of two important natural propulsive mechanisms (pure heaving and undulating) were carried out to elucidate and compare the features of thrust generation, power consumption, and efficiency as well as vortical patterns, by systematically varying the Strouhal and Reynolds numbers. This study helps to get insights into the behaviors of these mechanisms in producing the propulsive and stabilizing forces for engineering applications. The simulations were carried out in *Re* = 4000, 40000, and 400000, with the maximum trailing edge peak to peak amplitude of 0.2*L* and the Strouhal numbers in the range of [0.05, 2.5]. The time histories of the instantaneous inline and transverse hydrodynamic forces and moment, the time averaged inline force and consumed power, the efficiency, and the contours of vorticity and *Q* criterion were presented and discussed.

In general, the time dependent forces and moments show two peaks corresponding to the forward and backward strokes of the foil tip, in each cycle. The upstroke and downstroke peaks of the heaving and undulating mechanisms are similar at low St, but the difference between them considerably increases with increasing St and a greater value for heaving motions. Strouhal numbers of oscillations determine whether the motions are drag, neutral, or thrust dominated. Generally, the oscillation mechanisms are drag and thrust dominated, respectively, at low and high Strouhal numbers. The Strouhal number in which the transition from the drag dominated regime to the thrust dominated regime occurs is called “steady state Strouhal number.” The neutral or steady state Strouhal numbers of heaving St_*H*_^∗^ and St_*U*_^∗^ undulations in which the transitions from drag dominated regime to thrust dominated regime occur are St_*H*_^∗^ = 0.23 and St_*U*_^∗^ = 0.5 in *Re* = 4000, St_*H*_^∗^ = 0.05 and St_*U*_^∗^ = 0.25 in *Re* = 40000, and St_*H*_^∗^ < 0.01 and St_*U*_^∗^ = 0.09 in *Re* = 400000.

The curves of inline force (thrust) versus St are monotonously descending, according to the direction of the *x*-axis. However, the inline force curve corresponding to the undulating foil descends with a lower slope. Likewise, the curves of consumed power versus St are also monotonously descending and the curve corresponding to the undulating foil descends with a lower slope. The efficiency curves show firstly the ascending and then descending behaviors with respect to St. The peak efficiency points occur at St_*H*_^PE^ = 0.5 and St_*U*_^PE^ = 1.0 in *Re* = 4000, St_*H*_^PE^ = 0.05 and St_*U*_^PE^ = 0.5 in *Re* = 40000, and St_*H*_^PE^ = 0.01 and St_*H*_^PE^ = 0.25 in *Re* = 400000. The peak propulsive efficiency values, *η*_*H*_^*P*^ and *η*_*U*_^*P*^ for the heaving and undulating foils, respectively, are *η*_*H*_^*P*^ = 0.24 and *η*_*U*_^*P*^ = 0.4 in *Re* = 4000, *η*_*H*_^*P*^ = 0.27 and *η*_*U*_^*P*^ = 0.47 in *Re* = 40000, and *η*_*H*_^*P*^ = 0.22 and *η*_*U*_^*P*^ = 0.49 in *Re* = 400000. At low Strouhal numbers, the values of efficiency for heaving oscillations are greater than undulating oscillations. However, after the peak efficiency point of the undulating foil, at higher Strouhal numbers, the efficiency of the undulatory oscillations reaches higher values than the heaving oscillations.

Comparative observational features of the vortical patterns of both undulating and heaving foils show that the wake pattern of each motion depends on the Strouhal number. At low Strouhal numbers, the downstream wakes in the closely near regions of the foils' trailing edges show more similarities to each other. Moreover, at low St, the distinct vortices are formed with a delay. At higher Strouhal numbers, stronger vortices are produced and they are shed into the downstream at the tip of the trailing edge for both undulating and heaving foils. The formation of the vortices is directly related to the fluctuations of the pressure coefficients. The jumps in pressure coefficients represent high pressure gradient regions in which the vortices shed from the surface of the foil. The most significant difference between the pressure coefficient graphs and vortical patterns of the heaving and undulating foils is appearance of leading edge and surrounding vortices in the wake of the heaving foil which could considerably affect the power consumption and efficiency as well as the trace of oscillations. In addition, the *Q* criterions confirm our findings about the vortical patterns.
